# A New Function of *MbIAA19* Identified to Modulate *Malus* Plants Dwarfing Growth

**DOI:** 10.3390/plants12173097

**Published:** 2023-08-29

**Authors:** Jian Wang, Li Xue, Xiao Zhang, Yali Hou, Ke Zheng, Dongxu Fu, Wenxuan Dong

**Affiliations:** College of Horticulture, Shenyang Agricultural University, Shenyang 110866, China; botelongma@163.com (J.W.); lixue@syau.edu.cn (L.X.); zhangxiao8866@syau.edu.cn (X.Z.); houyali@syau.edu.cn (Y.H.); zhengke19870430@163.com (K.Z.); f18547590930@163.com (D.F.)

**Keywords:** Aux/IAA, auxin, dwarf, *Malus baccata*, transcriptome

## Abstract

The primary determinants of apple (*Malus*) tree architecture include plant height and internode length, which are the significant criteria for evaluating apple dwarf rootstocks. Plant height and internode length are predominantly governed by phytohormones. In this study, we aimed to assess the mechanisms underlying dwarfism in a mutant of *Malus baccata*. *M. baccata* dwarf mutant (*Dwf*) was previously obtained through natural mutation. It has considerably reduced plant height and internode length. A comparative transcriptome analysis of wild-type (WT) and *Dwf* mutant was performed to identify and annotate the differentially expressed genes responsible for the *Dwf* phenotype using RNA-seq and GO and KEGG pathway enrichment analyses. Multiple DEGs involved in hormone signaling pathways, particularly auxin signaling pathways, were identified. Moreover, the levels of endogenous indole-3-acetic acid (IAA) were lower in *Dwf* mutant than in WT. The Aux/IAA transcription factor gene *MbIAA19* was downregulated in *Dwf* mutant due to a single nucleotide sequence change in its promoter. Genetic transformation assay demonstrated strong association between *MbIAA19* and the dwarf phenotype. *RNAi-IAA19* lines clearly exhibited reduced plant height, internode length, and endogenous IAA levels. Our study revealed that MbIAA19 plays a role in the regulation of dwarfism and endogenous IAA levels in *M. baccata*.

## 1. Introduction

Dwarfing and tightly separated planting are the primary modes of modern fruit farming practices. Over the past century, extensive application of dwarfing rootstocks has led to increased planting density and production of fruits, even during early years of orchard development [1,2]. The complex features of plant dwarfism are regulated by multiple genes, such as *PcPIN1*, *OsBR6ox*, *PsGA3ox1*, *WRKY*, and *GRAS* [3,4,5,6,7]. Multiple mutants with dwarfing associated with phytohormones were uncovered, such as rice, maize, and apple, and the underlying gene functions were confirmed [8,9,10]. Phytohormones act as central players in the regulation of plant growth and development, in which auxin is the most significant signaling molecule because it is involved in almost all aspects of plant life. The development and phenotype of several organs could be influenced by auxin, including the root system, plant height, leaf shape, and reproductive organs, resulting in cell division and cell expansion at various stages of tissue development [11,12,13,14]. Clarifying the association between auxin and plant phenotypes is essential for understanding the mechanism of dwarfism in plants.

Auxins modify the expression of downstream genes that encode proteins involved in a wide range of physiological networks in plants [15,16,17]. Extensive studies have reported that auxins regulate the expression of downstream genes through ubiquitin-dependent proteolytic signal transduction system. First, transport inhibitor response1 (TIR1) protein forms a multi-subunit SCF ubiquitin ligase. Further, the complex interacts with auxin/indole-3-acetic acid (Aux/IAA), which is a transcriptional repressor that inhibits the expression of early auxin-responsive genes, eventually leading to the degradation of Aux/IAA proteins by the proteasome [18]. The affinity of TIR1 for Aux/IAA is influenced by the concentration of cellular auxin. High concentration of auxin induces the degradation of Aux/IAA; however, low concentration of auxin reduces the interaction between TIR1 and Aux/IAA [19]. Aux/IAA inhibits the transcriptional activity of auxin responsive factors (ARFs), which regulate the expression of genes that respond to auxin, by directly interacting with them. Thus, Aux/IAAs are crucial for controlling auxin-mediated activities [20,21].

Aux/IAA repressor proteins contain four highly conserved domains (I–IV). Of these, domain II, also known as the degron motif, is primarily responsible for the auxin-dependent degradation of Aux/IAAs [22]. Gain-of-function mutations in domain II of the Aux/IAA in *Arabidopsis thaliana* provided information on the role played by these proteins in modulating auxin responses and plant developmental processes. These mutations may reduce or abolish the interactions among TIR1 and Aux/IAAs. Many auxin-related developmental abnormalities, such as altered lateral root formation, stem hypocotyl elongation, leaf expansion, apical dominance, phototropism, and gravitropism, are displayed by these Aux/IAA gain-of-function mutants [23,24,25,26,27]. Genetic studies have revealed various morphological phenotypes in association with *Aux*/*IAA* genes in other plant species. Fewer crown and lateral roots were produced in rice (*Oryza sativa*) due to the negative regulation of auxin-regulated root development by *Osiaa9* [28]. Tomato (*Solanum lycopersicum*) plants with *SlIAA15* silencing exhibited reduced apical dominance and number of trichomes; thick, dark-green leaves with thicker pavement cells; longer palisade cells; and spongy mesophyll cells that have a wider intercellular space [29]. Plants with *SlIAA9* silencing exhibited abnormally shaped leaves, enhanced stem elongation, and increased leaf vascularization [30]. *Brassica napus IAA7* gain-of-function mutant inhibited stem elongation due to the transcriptional repression of *EXPA5* genes [31]. The overexpression of *PtoIAA9m* in *Populus* significantly repressed the development of secondary xylem [32]. When *TaIAA15* was ectopically expressed in rice, plant height and leaf angle were reduced [33]. In apple, *MdIAA2* overexpression led to the formation of smaller fruit [34]. The overexpression of *PpIAA19* (from peach (*Prunus persica*)) in tomato altered the plant development and fruit shape and substantially lengthened the internodes [35]. *OsIAA23*-knockout genotypes in rice exhibited significantly higher dwarfing and abnormalities in lateral root development [36]. *Aux*/*IAA* genes are extensively involved in determining plant height and regulating many other phenotypes. They can act as promoters or suppressors.

Apple (*Malus domestica*) is one of the most important commercial fruits. Generating dwarfing rootstocks and apple cultivars is important for improving economic yield and efficient orchard management. NIAB East Malling Research Station, England, conducts breeding to obtain dwarf rootstocks; after a century of development, many locally adapted dwarf rootstocks of apple were generated, such as M9, M26, and GC lines [37,38]. In northern China, *M. baccata* is frequently used as an apple rootstock because of its strong tolerance to cold and drought stresses. In this study, we aimed to assess the mechanisms underlying dwarfing in apple. We characterized an *M. baccata* dwarf mutant (*Dwf*), which exhibits shorter stature and internode lengths than the wild type (WT). RNA-seq, metabolites analysis, and extensive biological experiments were performed to reveal a novel function of *MbIAA19* associated with the phenotype of *Dwf*. This study provided a foundation for clarifying the relationship among *MbIAA19*, auxin, and dwarfism. Understanding the mechanism of dwarfism in *Dwf* will help in breeding rootstocks with better adaptability to various stresses.

## 2. Results

### 2.1. Dwf Mutant Exhibited Variation in the Phenotype of Multiple Organs

**Figure 1:**
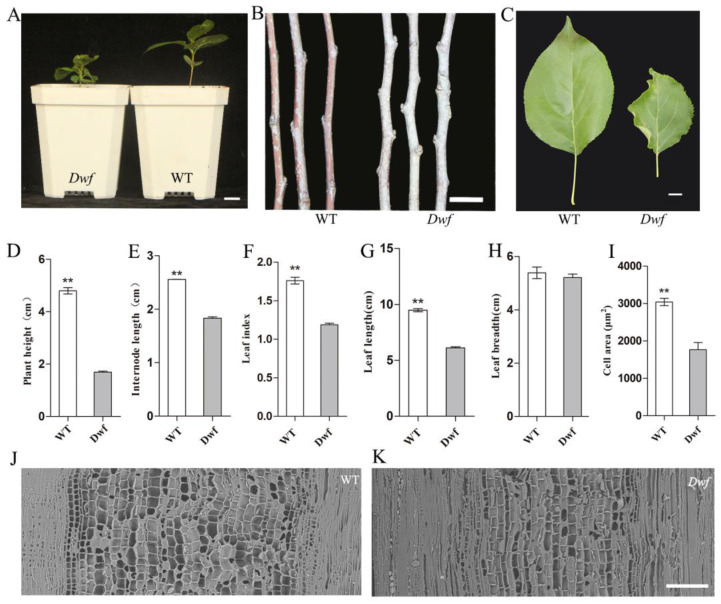
Morphological characterization of *Dwf* mutant. (**A**) Universal morphology of the dwarf mutant (*Dwf*) and wild type (WT) plants of *M. baccata* after 1 month of culture. Scale bar = 1 cm. (**B**) Annual branches of WT and *Dwf* plants. Scale bar = 1 cm. (**C**) Apical leaves of the annual branches of WT and *Dwf* plants. Scale bar = 1 cm. (**D**) Plant heights of WT and *Dwf* plants. (**E**) Internode lengths of the annual branches of WT and *Dwf* plants. (**F**) Calculation of the leaf length to width ratio of the WT and *Dwf* plants. (**G**) Leaf length of WT and *Dwf* plants. (**H**) Leaf breadth of WT and *Dwf* plants. (**I**) Cell areas of stem vessels in longitudinal sections. (**J**,**K**) Scanning electron microscopic observation of the longitudinal section of stems in annual branches of WT and *Dwf* plants. Scale bar = 0.2 mm. The asterisks above the error bars in (**D**–**G**,**I**) indicate significant differences at *p* < 0.01 using a one-way analysis of variance (ANOVA) and Dunnett’s test.

The plant height and internode length are important indicators of dwarfism. The spontaneous dwarf mutant of *M. baccata*, namely, the *Dwf* mutant, exhibited shorter plant height and internodes and lower leaf index than WT (Figure 1A–C). Observations revealed that compared with WT, *Dwf* mutant exhibited 1.6-fold shorter plant height, and its internode length, leaf index, and leaf length were reduced by 30% (Figure 1D,E), 34% (Figure 1F), and 38% on an average, respectively (Figure 1G). However, no discernible variation in leaf breadth was observed (Figure 1H). Surprisingly, *Dwf* mutant represents distinct curled leaves (Figure 1C).

The cytological differences in the two genotypes were studied using SEM. The cell area in the xylem regions of annual branches of the *Dwf* was 42% smaller than the WT (Figure 1I); it revealed that there were dramatically smaller longitudinal vascular bundles cells in the annual branches of the *Dwf* mutant than in the WT (Figure 1J,K).

**Figure 2:**
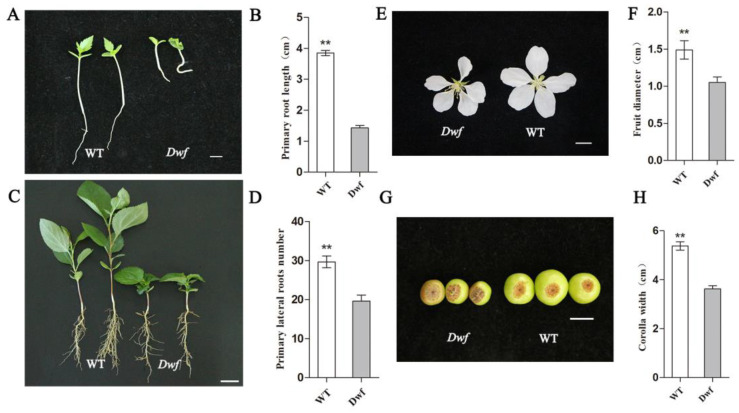
Morphological characterization of the root, flower, and fruit of the *Dwf* mutant. (**A**) Primary roots of one-week-old WT and *Dwf* plants. (**B**) Length of primary roots. (**C**) Lateral roots of WT and *Dwf* plants after 1 month of culture. (**D**) Quantitative analysis of primary lateral root number. (**E**) Flowers, (**F**) corolla width, (**G**) fruits, and (**H**) fruit diameter of WT and *Dwf* mutant. The asterisks above the error bars in (**B**,**D**,**F**,**H**) indicate significant differences at *p* < 0.01 using a one-way analysis of variance (ANOVA) and Dunnett’s test. Scale bar = 1 cm.

Furthermore, the root morphology, flowers, and fruits of *Dwf* mutant were significantly altered. It exhibited shorter primary roots (PR) (Figure 2A,B), fewer lateral roots (LR) (Figure 2C,D), and decreased corolla width and fruit diameter (Figure 2E–H).

### 2.2. Analysis of the DEGs on Phytohormone RNA-seq Data

To further understand the regulatory mechanism of dwarfism in *Dwf* plants, RNA-seq was performed to study the transcription profiles and identify the DEGs responsible for the *Dwf* phenotype. Young leaves at the tips of annual branches were collected from six individual plants (WT 1–3 and *Dwf* 1–3) for RNA-seq analysis. GO and KEGG pathway enrichment analysis revealed numerous genes associated with signal transduction via phytohormones and hormone response processes (Figure S1A,B). Compared with WT, 352 and 460 genes were up- and downregulated, respectively, in the *Dwf* plants (log2 ≥ 1) (Figure S1C). A total of 17 hormone-signaling- and growth-related genes were subjected to qRT-PCR to verify the DEGs (Figure S2A,B). The results of RNA-seq and qRT-PCR were consistent.

**Figure 3:**
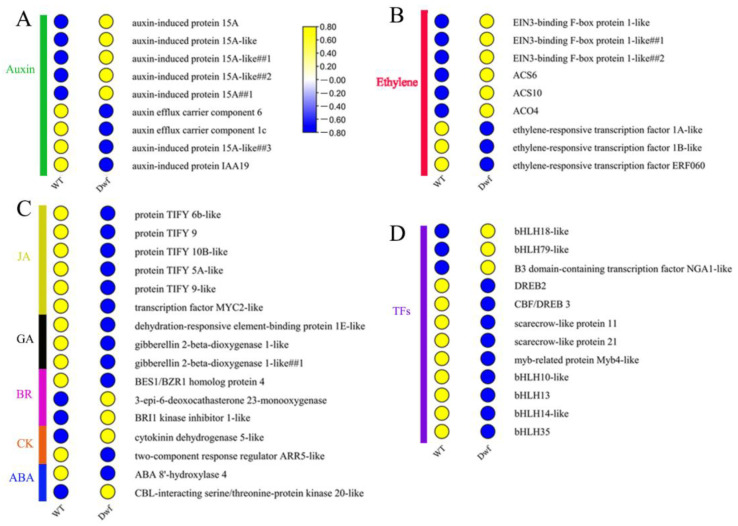
A heatmap of differentially expressed genes (DEGs) associated with phytohormone pathways. (**A**) DEGs associated with the signaling and biosynthesis pathways of auxin. (**B**) DEGs associated with the signaling and biosynthesis pathways of ethylene. (**C**) DEGs related to the signaling and biosynthesis pathways of JA, GA, BR, CK, and ABA. (**D**) Transcription factors involved in hormone signaling.

To thoroughly investigate the complete transcriptional pattern of DEGs related to phytohormones, DEGs associated with the signaling and biosynthetic pathways of IAA, ethylene (ET), jasmonic acid (JA), GA, brassinosteroids (BR), cytokinin (CK), and abscisic acid (ABA) were further functionally annotated. Crucial proteins involved in the auxin signal transduction pathway were altered. The auxin-responsive genes, including *MbAUX*/*IAA19* and *MbSAURs*, were overall downregulated (Figure 3A). *MbAUX*/*IAA19* is one of the major molecular components involved in auxin signaling, and *SAURs* regulate the cell expansion which has been demonstreated [39]. In addition, auxin efflux carrier components (*MbPIN1* and *MbPIN6*) were significantly downregulated in *Dwf* mutant (Figure 3A). DEGs associated with ET signaling such as *EIN3*-binding *F-box* genes (*MbEBF1s*), which physically interact with EIN3 and EIL1 proteins for degradation, were upregulated in *Dwf* mutant [40]. ET-biosynthesis-related genes (*MbACO4*, *MbACS6*, and *MbACS10*) were upregulated, whereas three ET-responsive transcription factors genes (*MbERF1A*, *MbERF1B*, and *MbERF060*) were downregulated in *Dwf* (Figure 3B). *MbTYFYs* (JAZs) involved in the JA signaling pathway were downregulated in *Dwf* mutant, the depletion of JAZ proteins is also associated with reduced growth and development [41]; MYC2, a key transcription factor (TF) of JA signaling pathway, was downregulated in *Dwf* mutant (Figure 3C).

GA, BR, CK, and ABA signaling pathways were altered in *Dwf* (Figure 3C). Among GA signaling genes, *MbDREB1E* (dehydration-responsive element-binding protein 1E) and *MbGA20OX1* (gibberellin 2-beta-dioxygenase 1; responsible for the bioactive transformation of GAs) were downregulated. Additionally, several genes related to signaling and biosynthesis of BR, CK, and ABA were altered. BRI1-EMS-SUPPRESSOR1/BRASSINAZOLE RESISTANT4 (BES1/BZR4) plays as key role in BR signaling, was downregulated in *Dwf*. Similarity, BKI1 act as a negative regulator of BR sinnalling and was up-regulated in *Dwf* (Figure 3C). Cytokinin oxidase/dehydrogenase5 (CKX5), a destructor of cytokinin, was up regulated in *Dwf* (Figure 3C). ABA 8′-hydroxylation act as the major components involved in catabolic pathway of ABA and was downregulated in *Dwf* (Figure 3C). TFs involved in hormone signaling were downregulated in *Dwf* plants (Figure 3D). These results demonstrate that the phytohormone signaling and biosynthetic pathways were affected in the *Dwf* mutant. Interestingly, 26% DEGs involved in phytohormone signaling were associated with the auxin signaling pathway. Therefore, auxin was observed to be an essential hormone in controlling plant growth in *Dwf* mutants.

### 2.3. Endogenous Auxin Levels Were Lower in Dwf Mutant

**Figure 4:**
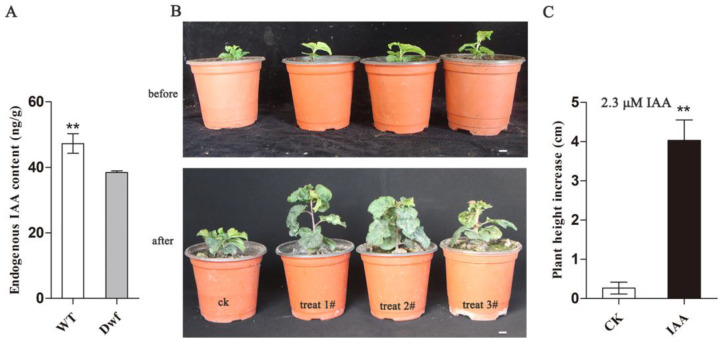
Analysis of endogenous auxin levels and exogenous auxin treatment. (**A**) Endogenous auxin levels in WT and *Dwf* mutant leaves. (**B**) *Dwf* mutant plants were sprayed with 2.3 μM IAA once a week for 4 weeks. Growth status of *Dwf* mutant before and after auxin treatment. (**C**) Plant height increased after treatment with 0.4 mg·L^−1^ IAA than after that with water for 1 month. The asterisks above the error bars indicate significant differences at *p* < 0.01 calculated using a one-way analysis of variance (ANOVA) and Dunnett’s test.

To investigate whether DEGs related to auxin signaling and biosynthesis pathways are associated with the change in endogenous auxin levels, endogenous IAA levels were detected in both genotypes. Endogenous IAA content was substantially lower in the leaves of the *Dwf* mutant than in those of the WT (Figure 4A). We investigated whether auxin deficiency in *Dwf* mutant affected the dwarfing. One-month-old *Dwf* seedllings were sprayed with 0.4 mg·L^−1^ IAA once a week for 4 weeks and plant growth was observed. Interestingly, plant height was significantly increased in the *Dwf* mutant after IAA treatment compared to after water treatment (Figure 4B,C). These findings suggested that the endogenous auxin levels were altered in the *Dwf* mutant, and this alteration played a key role in dwarfing.

### 2.4. Analysis of MbIAA19 Gene Expression and Promoter Sequence

**Figure 5:**
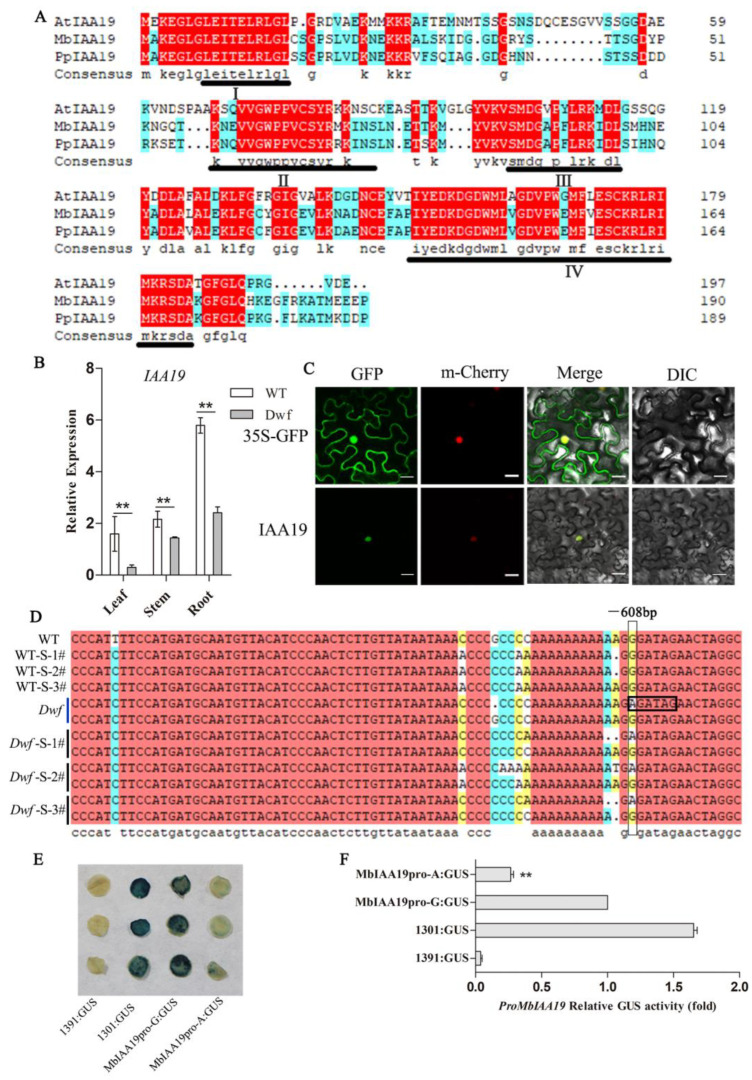
Analysis of *MbIAA19* gene expression and promoter sequence. (**A**) Amino acid sequence of MbIAA19 and alignment of MbIAA19 with IAA19 protein sequences from *Prunus persica* and *Arabidopsis thaliana*. Four conserved domains (I–IV) of IAA19 are shown. (**B**) Relative expression level of *MbIAA19* in different organs of *Dwf* plants compared with WT plants. (**C**) Subcellular localization analysis of MbIAA19. (**D**) Variations of alleles in the *MbIAA19* promoter between WT, *Dwf*, and seedllings (WT-S1-3# and *Dwf*-S1-3#), the black box represents the novel cis-acting element [(A/T)GATA(A/G)]. (**E**) The GUS activity of *MbIAA19* promoter in infected tobacco leaves, GUS:1301 and GUS:1391 served as positive and negative controls, respectively. (**F**) Quantitative assessment of relative GUS activity in infected tobacco leaves. Different colors in (**A**,**D**) represent sequences with different levels of conservation. The asterisks above the error bars in (**B**,**F**) indicate significant differences at *p* < 0.01 using a one-way analysis of variance (ANOVA) and Dunnett’s test.

*MbIAA19* is a member of the AUX/IAA gene family. Sequence analysis revealed that *MbIAA19* contains an open reading frame of 573 bp, encoding 190 amino acids; the *MbIAA19* sequences from WT and *Dwf* were identical. After aligning with IAA19 protein sequences from *P*. *persica* and *A. thaliana*, it was revealed that MbIAA19 proteins contain four conserved domains (domains I, II, III, and IV; Figure 5A). The expression of *MbIAA19* in several tissues was studied using qRT-PCR. It was significantly downregulated in the roots, stems, and leaves of *Dwf* plants (Figure 5B). Next, we performed a subcellular localization analysis of *MbIAA19*. In contrast to the 35S::GFP protein (which was identified in both the cytoplasm and nucleus when released alone in vivo in the leaf epidermal cells of tobacco), the GFP-IAA19 fusion proteins and mCherry protein were primarily located in the nucleus (Figure 5C). The sequences of the *MbIAA19* promoters in WT and *Dwf* mutant were compared to identify any mutation in the promoter region. A single nucleotide change from G to A (−608 bp) was observed in *Dwf*; it was a heterozygous mutation in *Dwf* mutant and seedlings with *Dwf* phenotype. The altered promoter creates a novel cis-acting element [(A/T)GATA(A/G)], a GATA transcription factor binding site. (Figure 5D). Further, GUS activity was analyzed to assess how variation in the alleles affects the activities of the *MbIAA19* promoter. Interestingly, altered promoters in tobacco leaves exhibited markedly reduced levels of GUS protein and GUS activity (Figure 5E,F and Figure S3). These findings suggest that *MbIAA19* is a candidate gene responsible for the *Dwf* mutant phenotype.

### 2.5. Downregulation of MbIAA19 Inhibited Plant Height and Internode Length in GL-3

**Figure 6:**
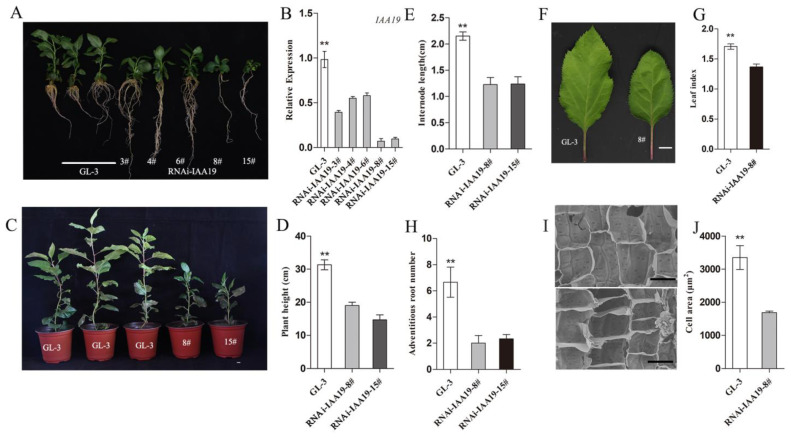
Morphological characterization of the *RNAi-IAA19* lines compared with GL-3. (**A**) Universal morphology of GL-3 and five *RNAi-IAA19* lines. (**B**) Expression level of IAA19 in GL-3 and five *RNAi-IAA19* lines. (**C**) GL-3 and *RNAi-IAA19* lines after grown in the incubator for 2 months. Scale bar = 1 cm. (**D**) Plant height of GL-3 and two *RNAi-IAA19* lines (8# and 15#). (**E**) Internode length of GL-3 and two *RNAi-IAA19* lines (8# and 15#). (**F**) Images of the leaves from the third leaves of 2-month-old seedlings of GL-3 and *RNAi-IAA19-8*#. Scale bar = 1 cm. (**G**) Comparison of the ratio of leaf length to width of *RNAi-IAA19* lines and GL-3. (**H**) Adventitious root numbers of GL-3 and two *RNAi-IAA19* lines (8# and 15#). (**I**) Scanning electron microscopic analysis of longitudinal segment of the stems of GL-3 (**up**) and *RNAi-IAA19-8*# (**down**), Scale bar = 40 μm. (**J**) Calculation of cell areas of the stems of GL-3 and *RNAi-IAA19-8*#. The asterisks above the error bars in (**B**,**D**,**E**,**G**,**H**,**J**) indicate significant differences at *p* < 0.01 using a one-way ANOVA and Dunnett’s test.

The function of *MbIAA19* and its role in growth regulation in *Malus* was confirmed using GL-3, the open-pollinated cultivar of *M. domestica* ‘Royal Gala’. GL-3 was transformed with the vector *RNAi-IAA19*. From all the regenerated lines, five distinct lines with *MbIAA19* downregulation (*RNAi-IAA19*) were obtained. The phenotypes of all five *RNAi-IAA19* plants mimicked the phenotype of *Dwf* mutant, with clearly reduced stature and fewer roots (Figure 6A). For the subsequent studies, two *RNAi-IAA19* lines (8# and 15#) with significant reduction in *MbIAA19* expression were selected (Figure 6B).

After cultivating in a greenhouse for 2 months, compared with GL-3 plants, plant height dramatically reduced by 34% and 49% in the *RNAi-IAA19* lines 8# and 15#, respectively. Their plant height was comparable with that of *Dwf* mutant (Figure 6C,D). The internode length was significantly reduced by approximately 43% in 8# and 15#; the variation in the internode length was wider than that in *Dwf* mutant (Figure 6E). The leaf index was lower than that of GL-3; it was similar to that of *Dwf* mutant, which exhibited shorter leaf length (Figure 6F,G). The *RNAi-IAA19* lines exhibited less adventitious roots (Figure 6H). According to SEM analysis, the cell areas of longitudinal cells were reduced by 54%. The effect of inhibition of cell expansion on dwarf plants was further determined (Figure 6I,J). Collectively, the results revealed that *MbIAA19* indeed corresponds to the *Dwf* phenotype.

### 2.6. Endogenous Auxin Levels Were Lower in the Leaves of RNAi-IAA19-8# Than in Those of GL-3

**Figure 7:**
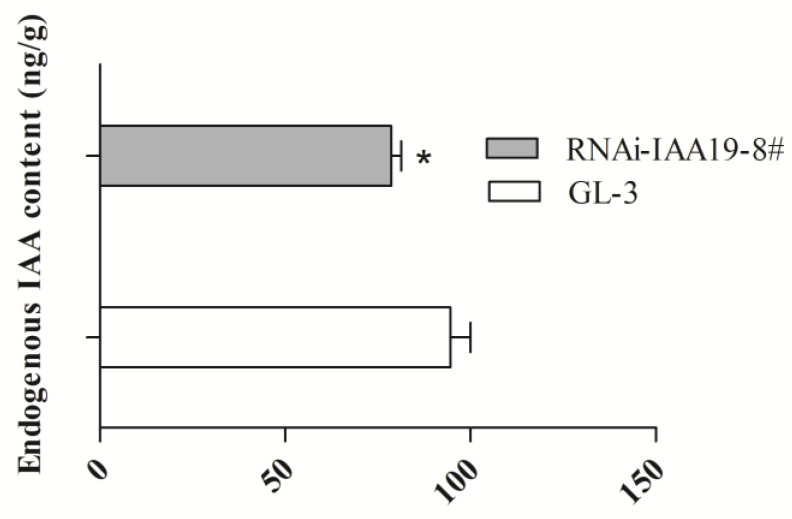
Endogenous auxin levels in GL-3 and *RNAi-IAA19-8#* leaves. The asterisks above the error bars indicate significant differences at *p* < 0.05 using a one-way ANOVA and Dunnett’s test.

To investigate whether the endogenous IAA levels were influenced by the expression level of *MbIAA19*, the endogenous IAA levels were assessed in GL-3 and *RNAi-IAA19-8*# leaves. The endogenous IAA level in *RNAi-IAA19-8*# leaves was substantially lower than that in GL-3 leaves (Figure 7). This confirmed that the changes in *MbIAA19* expression are highly associated with the *Dwf* phenotype.

## 3. Discussion

Studies on the development of dwarfing rootstocks and understanding the mechanisms underlying dwarfing are ongoing in apple. Many excellent dwarfing rootstocks have been developed [42]. Numerous dwarf lines of *Malus* have been discovered [10,43,44], and widely accepted standards for evaluating dwarfing rootstocks have been established [38,45]. Additionally, some studies have attempted to explain the mechanism of occurrence of dwarf phenotypes from various perspectives, including the role of phytohormones and nutrient metabolism [46,47]. In this study, the *Dwf* mutant exhibited phenotypic changes in various organs (Figure 1 and Figure 2). From the results, it can be concluded that the inhibition of vessel cell expansion causes the dwarfism in *Dwf* mutant (Figure 1I); however, due to the hypodevelopment of vessels, a potential decrease in nutrient transport capability can also be a cause of dwarfism.

Previous studies have revealed that phytohormones are important factors that regulate dwarfism. Increase in the endogenous or exogenous concentrations of auxin [11,48], gibberellins [49], and brassinosteroid [50] promotes the plant height during suitable levels. The *Dwf* mutant exhibits low concentrations of endogenous IAA (Figure 4A), which is consistent with previous studies [11,48]. RNA-seq revealed several DEGs related to auxin, ET, gibberellin, and brassinolide signaling in *Dwf* mutant (Figure 3A–C). However, no significant DEG related to auxin biosynthesis was identified in *Dwf* mutant (Figure S4). Therefore, the reason underlying the lower endogenous auxin levels should be identified.

As one of major molecular components families (Aux/IAAs, ARFs, ABP1 and TIR1) involved in auxin signaling, increasing the stability of Aux/IAA proteins leads to the disruption or obstruction of auxin signaling, which could even lead to a mutation that was unresponsive to auxin [22,51]. We concluded that the majority of *Aux*/*IAA* family members have multiple roles in regulating morphogenesis with regard to the development of plant organs. As a result, the *Aux*/*IAA* gene family may be a suitable candidate and a starting point to understand the mechanism of multi-organ variation during evolution. However, *Aux*/*IAA* members are rarely studied in the *Malus* species. Previous studies on Aux/IAA in *Malus* primarily focused on stress resistance, and little attention has been paid to fruit development and plant phenotypes [52,53,54]. *MbIAA19* was the only major component involved in auxin signaling identified by RNA-seq with a significant difference between WT and *Dwf*, which was uniformly downregulated in different organs of *Dwf* (Figure 5B). Changes in auxin content stimulate our interest in the relationship between auxin accumulation and signaling because numerous studies have demonstrated the essential feedback regulation between auxin accumulation and signaling pathway; auxin affects the activation of signaling components, while Aux/IAA-ARF affect the accumulation of auxin through further regulating the expression of *PINs* [55,56]. Sequence analyses were used to investigate the sequence changes that may affect gene expression of *IAA19*; fortunately, a single nucleotide change in the promoter of *MbIAA19* resulted in a novel cis-acting element [(A/T)GATA(A/G)], a GATA transcription factor binding site [57,58]. It is tempting to speculate that reduced promoter activity (Figure 5D–F) was because of a transcriptional repressor binding to the novel element. These results prompted us to investigate if *MbIAA19* is involved in determining the dwarfing phenotype in *Dwf* mutant. In this study, *RNAi-IAA19* lines were generated in an attempt to promote the studies on morphological regulation related to the Aux/IAA members in *Malus*.

The inhibition of plant height, internode elongation, and root development by the downregulation of *MbIAA19* was confirmed in two stable *RNAi-IAA19* transgenic lines. This indicated that *MbIAA19* may act across multiple tissues (Figure 6). However, in addition to shorter stems, smaller leaves, and fewer roots, the *Dwf* mutant also exhibits variation in leaf shape, corolla width, and fruit diameter (Figure 1C and Figure 2E–H). It is unclear whether these phenotypes related to the reproductive organs will manifest in the *RNAi-IAA19* lines. Similar to the *Dwf* mutant, low concentrations of endogenous IAA were exhibited in *RNAi-IAA19* lines (Figure 7). This indicated that *MbIAA19* is related to *Dwf* phenotype and has an impact on endogenous auxin content. The known phenotypic variations in *RNAi-IAA19* did not precisely match with those in *Dwf* mutant, e.g., curled leaves were not observed, suggesting that *MbIAA19* may not be the only gene responsible for the variation in *Dwf* mutant phenotype. Therefore, further studies are needed to assess other gene families responsible for the dwarfing mechanism in *Dwf* mutant. Overall, *MbIAA19* regulates the morphology of multiple organs, and the phenotypes of developmental repression are related to the inhibition of *IAA19* expression. Sequence analysis indicated that the closest homolog to *MbIAA19* in *A. thaliana* is *AtIAA19*/*MSG2*. In *A. thaliana*, *AtIAA19* is involved in the regulation of stamen filament development, plays a key role in the formation of roots, and reveals defects in tropic responses in hypocotyls [59,60,61,62]. Indeed, the *RNAi-IAA19* lines and msg2 mutant exhibited a reduced number of lateral roots (Figure 6I) [61]. The *RNAi-IAA19* lines exhibited significant changes in plant height, internode length, and leaf shape in *Malus* (Figure 6B,E–H), which were not reported in previous studies. Differences in the expression of *MbIAA19* did not cause defective tropic responses, indicating that functional divergence occurred between *Malus* and *A. thaliana*. *PpIAA19* and *VvIAA19* function as growth promoters for plant height; over-expressing *PpIAA19* in tomato (*Solanum lycopersicum* cv. ‘Micro-Tom’) indicated that *PpIAA19* was involved in promoting root length and stem elongation, inhibiting fruit development and fertilization [35]; over-expression of *VvIAA19* in *A. thaliana* also had a notable effect on plant growth, which exhibited higher plant and longer roots then the WT [63]. The above results suggested a new function of *MbIAA19* to modulate *Malus* plants, dwarfing growth.

In conclusion, we identified a differentially expressed transcription factor gene in the *Dwf* mutant. This mutant exhibited lower endogenous IAA level and stable downregulation of *MbIAA19*. A single nucleotide variation in the promoter of *MbIAA19* in *Dwf* resulted in lower promoter activity than that in WT. The downregulation of *MbIAA19* inhibited plant height and internode length. The endogenous IAA levels were lower in *RNAi-IAA19* lines as well, demonstrating the association between *MbIAA19* and the phenotype of *Dwf* mutant. In the future, it will be interesting to clarify how *MbIAA19* is associated with endogenous IAA levels.

## 4. Materials and Methods

### 4.1. Plant Material

*Dwf* mutant, the dwarf mutant of *M. baccata*, was obtained through a natural mutation of *M. baccata* from Hulunbeier League, Inner Mongolia (48.01363 N, 122.73757 E) in China. Previous genetic analyses have suggested that the dwarfing trait of *Dwf* is a quality trait controlled by a dominant heterozygous gene [64]. Therefore, the seedllings of *Dwf* were of two types, dwarf and wild type in 1:1 ratio. Fortunately, the dwarf plants exhibit extremely similar multi-organ linkage variation in the roots, stems, leaves, flowers, and fruits, providing sufficient materials for the successful execution of this study. This study included three WT (WT 1–3) and three *Dwf* plants (*Dwf* 1–3). After vernalization, the seeds collected from *Dwf* plants were sown in soil. The plants were grown in a smart incubator at 25 °C with 12 h:12 h (light: dark cycle) and 70% relative humidity. To establish in vitro shoot proliferation in GL-3 (the open-pollinated cultivar of *M. domestica* ‘Royal Gala’), MS media containing 3% sucrose and 0.7% agar (*w*/*v*) supplemented with 0.3 mg L^−1^ 6-benylaminopurine (6-BA), 0.2 mg L^−1^ indole acetic acid (IAA), and 0.1 mg L^−1^ gibberellic acid (GA3) were used.

### 4.2. Phenotypic and Cytological Analyses

The botanical traits investigated in this study included plant height, internode length, leaf index, primary roots length, primary lateral root number, corolla width, and fruit diameter. The leaf index was calculated as the leaf length to width ratio. One-month-old seedllings were used to measure plant height, whereas two-month-old seedllings were used to measure the changes in plant height after IAA treatment. The primary root length was measured using 1-week-old seedllings. The internode lengths were analyzed using the annual branches of WT and *Dwf* plants. The leaf length and width, root length, fruit diameter, and corolla were measured using a vernier caliper. At least three biological replicates were used for the analysis, and the average of each index was calculated.

Scanning electron microscopy (SEM; HITACHI Regulus 8100, Hitachi, Tokyo, Japan) was performed to study the stems of WT and *Dwf* plants. The third internode of annual branches was prepared as follows. The internodes were rinsed three times with PBS after fixing in 2.5% glutaraldehyde. Further, the samples were dehydrated using ethanol gradients and substituted with tertiary butyl alcohol. After they were dried (VFD-30) and sprayed with gold (MC1000), the images were acquired using SEM. ImageJ2 software (NIH, Bethesda, MD, USA) was used to measure the cellular areas in the longitudinal section of vessels of stem.

### 4.3. RNA-seq Analysis and Gene Expression

Total RNA was extracted from the young leaves of WT 1–3 and *Dwf* mutant 1–3. RNA sequencing libraries were constructed and sequenced on an Illumina NovaSeq 6000 system (Illumina, San Diego, CA, USA). RNA-seq data from the two genotypes were analyzed according to the Apple Genome (GDDH13 Version 1.1, https://iris.angers.inra.fr/gddh13/the-apple-genome-downloads.html, accessed on 5 June 2017). Quality control and trimming processes were performed as previously described [65]. Fragments per kilobase of transcript per million mapped read (FPKM) values were estimated to analyze the differentially expressed genes (DEGs) between the two genotypes. The genes were considered to be significantly expressed if the absolute fold change was ≥1 and *p* was <0.01 as determined by an R package (R Foundation for Statistical Computing, Vienna, Austria). Gene Ontology (GO, http://geneontology.org/, accessed on 1 May 2019) and Kyoto Encyclopedia of Genes and Genomes (KEGG, http://www.kegg.jp/kegg, accessed on 1 May 2019) pathway enrichment analyses were performed to annotate the DEGs. All raw and processed RNA-seq data from this study have been released on SRA Run Selector, with the SRA accession number PRJNA543379 (NCBI BioProject, https://www.ncbi.nlm.nih.gov/Traces/study/?acc=PRJNA543379, accessed on 15 June 2020).

Total RNA of leaves extraction was conducted using CTAB as described previously [66]. The cDNA was generated using PrimeScriptTM RT reagent Kit with gDNA Eraser (TaKaRa, Dalian, China). The TB Green^®^ Premix Ex TaqTMII (TaKaRa) and ABI QuantStudio 6 Flex instrument (Applied Biosystems, Waltham, MA, USA) were used for quantitative PCR (qPCR) analyses. Reaction system and qPCR process were conducted as described previously [66]. RNA extracted from each plant was used as one biological replicate, and a total of three technical and biological replicates to calculate the variances of population (SD). The expression levels were standardized with those of the *18S* genes. Specific primers are listed in Supporting Information (Table S1).

### 4.4. Determination of the Contents of Endogenous IAA

The contents of endogenous IAA were determined as previously described [67] using 0.5 g young leaves of WT and *Dwf* mutant plants. The experiment involved three biological and technical replicates. [^13^C]_6_-IAA (OlChemIm Company, Olomouc, Czech Republic) was used as the internal standard.

### 4.5. Exogenous IAA Treatment

One-month-old seedllings with *Dwf* phenotype were sprayed with 0.4 mg·L^−1^ IAA once a week for 4 weeks, spraying with water as control. The experiment was performed in three biological replicates.

### 4.6. Cloning, Plasmid Construction, and Genetic Transformation

The full-length sequence of the promoter and sense strand of *MbIAA19* (MD17G1198100) was searched in the genomics database for the Rosaceae family (GDR, https://www.rosaceae.org/, accessed on 7 August 2017). The full length 1600-bp promoter sequence of WT and *Dwf* mutant plants was cloned into the binary vector pCAMBIA1391 for sequencing and GUS activity analysis. The CDSs were subcloned from the leaves of *Dwf* mutant plants into *35S::GFP* to create *35S::IAA19:GFP*, and antisense partial sequences were amplified to create antisense suppression vectors (*RNAi::IAA19*). Vectors were constructed using ClonExpress II One Step Cloning Kit (C115, Vazyme, Nanjing, China). The primers are listed in Supporting Information (Table S1).

### 4.7. Confocal Microscopy and GFP and GUS Analyses

Constructed vectors were introduced into *Agrobacterium tumefaciens* GV3101, which was used to infect the leaves of 4-week-old tobacco (*Nicotina benthamiana*) plants. Green fluorescent protein (GFP) and red fluorescent protein (mCherry) were observed using a laser scanning confocal microscope (TCS SP8, Leica, Wetzlar, Germany). The excited points of GFP and mCherry were selected as previously [68]. For detecting GUS activity in the infected tobacco leaves, GUS staining was performed. The infected leaves were incubated with X-Gluc buffer for 48 h at 37 °C and soaked in ethanol to remove chlorophyll. Further, fluorometric assays were performed for assessing GUS activity as previously described [69].

### 4.8. Plant Transformation

The *RNAi::IAA19* vector was introduced into the *A. tumefaciens* strain EHA105. The young leaves of in vitro-grown “GL-3” were used for transformation. The transformed bacterial cells were incubated in LB medium at 28 °C, 180 rpm for 12–15 h, then diluted to OD_600_ = 0.5 in MS medium containing 1.5% sucrose and 0.5% glucose (*w*/*v*) supplemented with 100 μM acetosyringone (AS). The young leaves were cut into segments 3 mm wide, then the segments were gently shaken in bacterial suspension for 8 min. After transformation, the segments were transferred into co-culture medium for 3 d in the dark. Then the segments were cultivated on a selective medium containing 50 mg·L^−1^ kanamycin (Kan), 250 mg·L^−1^ cefixime (Cef) and 250 mg·L^−1^ timentin (Tim) for a few months, which were changed every half a month. The incubation was in the conditions of room temperature 25 °C and a photoperiod of 16 h:8 h (light:dark). Finally, mediums were configured as described by Dai et al. [70].

### 4.9. Statistical Analysis

The significant differences in the phenotypic data, gene expression, IAA production, and GUS activity were assessed using one-way analysis of variance (ANOVA) and Dunnett’s test (** *p* < 0.01, * *p* < 0.05) as specified in the figure legends. Each sample was evaluated at least three times.

## Data Availability

All the raw and operated RNA-seq data applied in this work have been released on SRA Run Selector, with the SRA accession number PRJNA543379 (NCBI BioProject, https://www.ncbi.nlm.nih.gov/Traces/study/?acc=PRJNA543379, accessed on 15 June 2020).

## Supplementary Materials

The following supporting information can be downloaded at: https://www.mdpi.com/article/10.3390/plants12173097/s1, Figure S1: DEGs between the WT and *Dwf* mutant; Figure S2: Confirmation of the DEGs by quantitative real time-PCR (qRT-PCR); Figure S3: The GUS activity of MbIAA19 promoter in tobacco leaves. Figure S4: A heat map of seven TAR genes (tryptophan aminotransferase-related gene) and nine YUCCA genes (indole-3-pyruvate monooxygenase gene) in the WT and *Dwf* identified using RNA-seq. Table S1: Primers used in this study.
