# Supplementary material for: A New Function of MbIAA19 Identified to Modulate Malus Plants Dwarfing Growth
Source: Plants (Basel). 2023 Aug 29;12(17):3097. doi: 10.3390/plants12173097 (PMC10490418; doi:10.3390/plants12173097)
Supplement: Supplementary file 1 [file plants-12-03097-s001.zip › plants-2526984-supplementary.pdf]

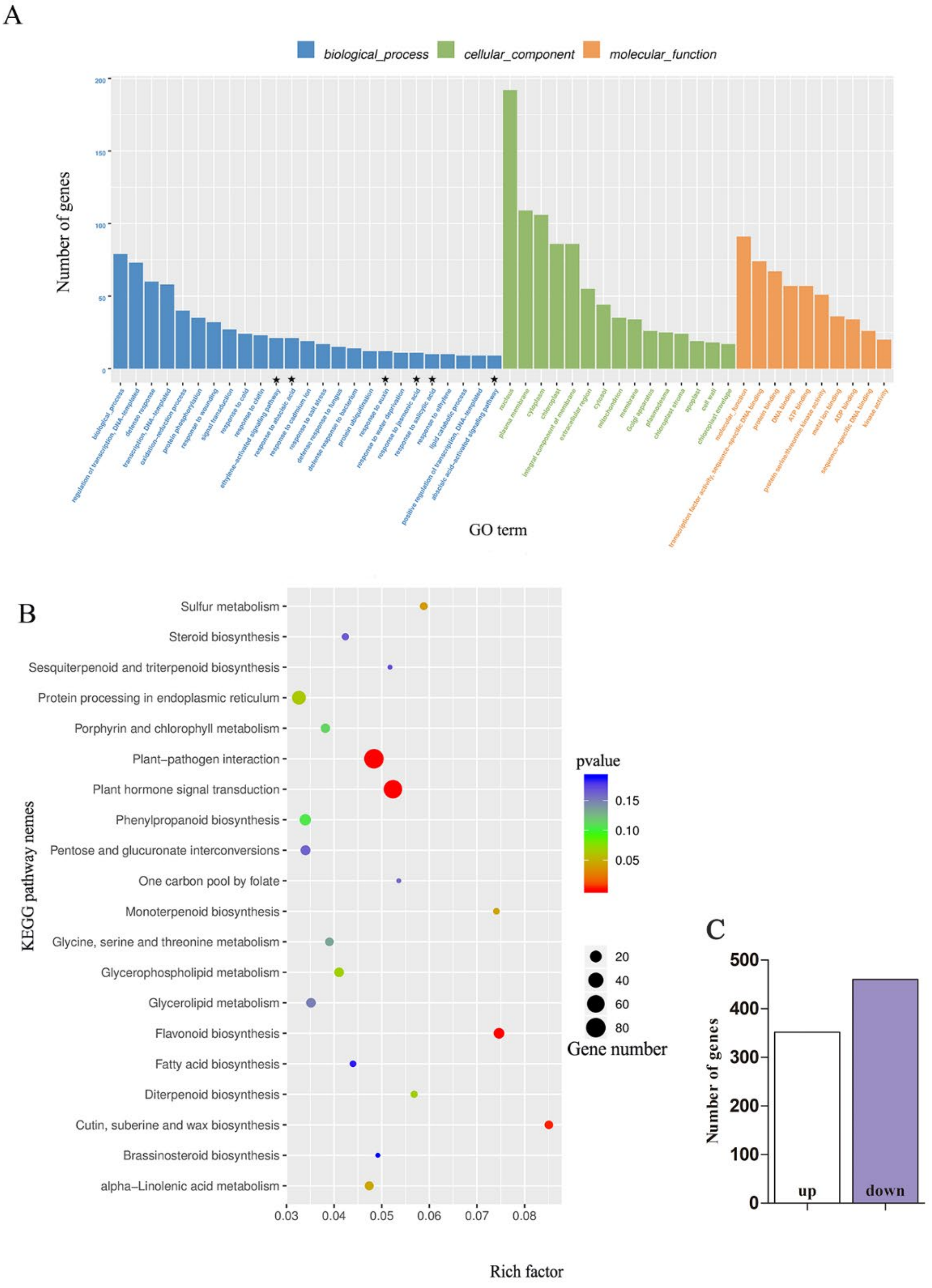

Figure S1. DEGs between the WT and *Dwf* mutant.

(A) GO term enrichment analysis of the DEGs, '★' indicates that the hormones are associated with a biological process. (B) KEGG pathway database enrichment analysis of the DEGs. (C) Number of up-regulated and down-regulated genes between the WT and *Dwf* mutant.

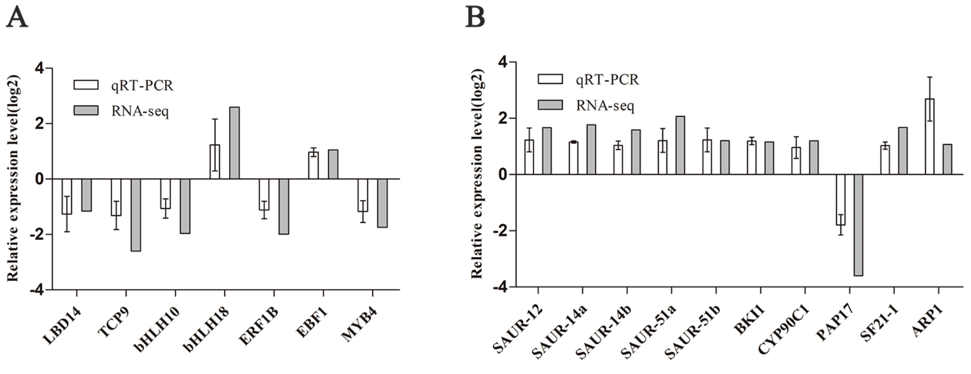

Figure S2. Confirmation of the DEGs by quantitative real time-PCR (qRT-PCR). A total of 17 DEGs obtained from RNA-seq analysis were selected for qRT-PCR validation. (A) Seven transcription factors and growth-related genes. (B) Ten genes related to hormone-signaling pathway. Log2FC values of RNA-seq and qRT-PCR are presented.

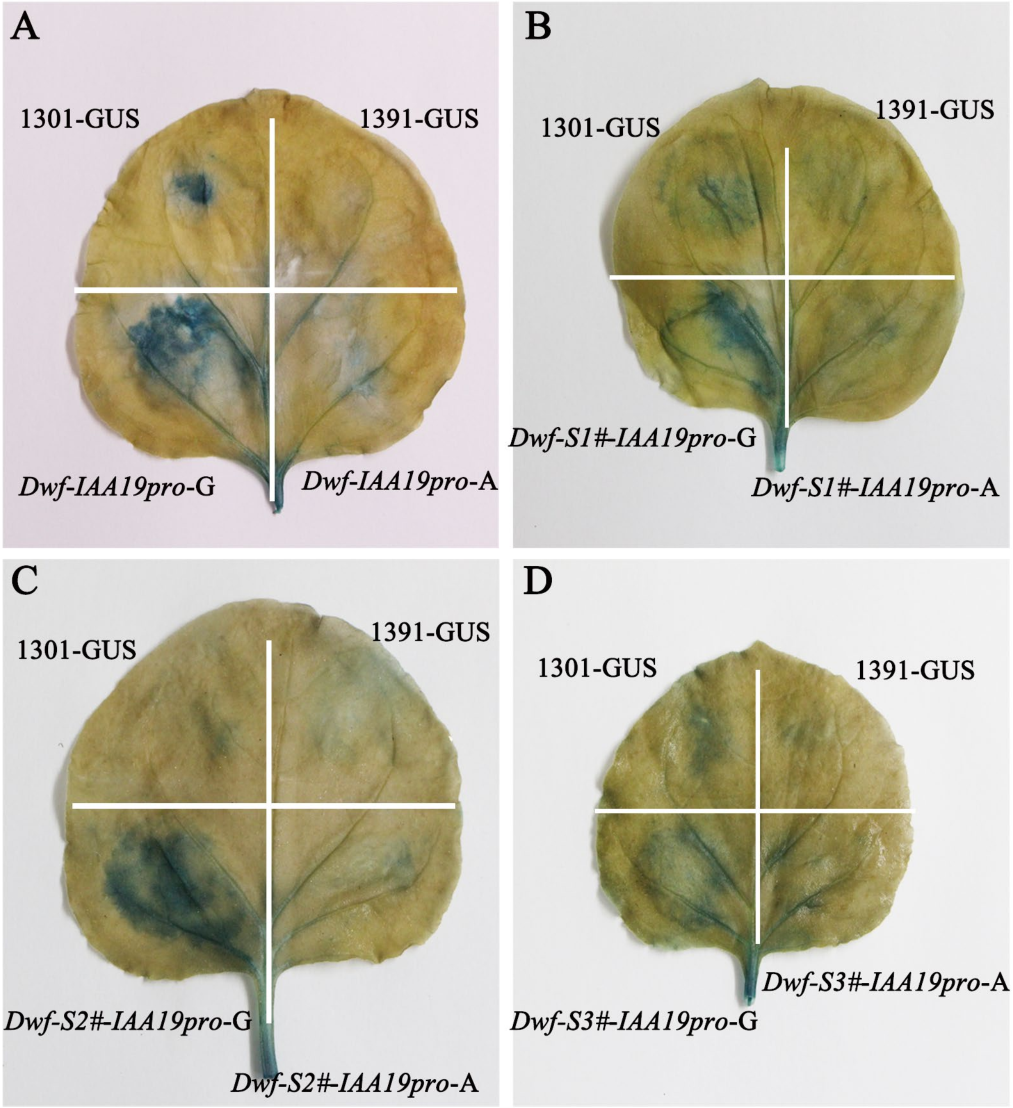

Figure S3. The GUS activity of *MbIAA19* promoter in tobacco leaves.

(A) The staining level of GUS protein in tobacco leaves, initiated by the altered promoters of *MbIAA19* in *Dwf*. (B) The staining level of GUS protein in tobacco leaves, initiated by the altered promoters of *MbIAA19* in *Dwf* seedllings 1# (*Dwf-S1#*). (C) The GUS protein level in tobacco leaves, initiated by the altered promoters of *MbIAA19* in *Dwf* seedllings 2# (*Dwf-S2#*). (D) The GUS protein level in tobacco leaves, initiated by the altered promoters of *MbIAA19* in *Dwf* seedllings 3# (*Dwf-S3#*). The staining level represents the abundance of GUS protein in the leaves.

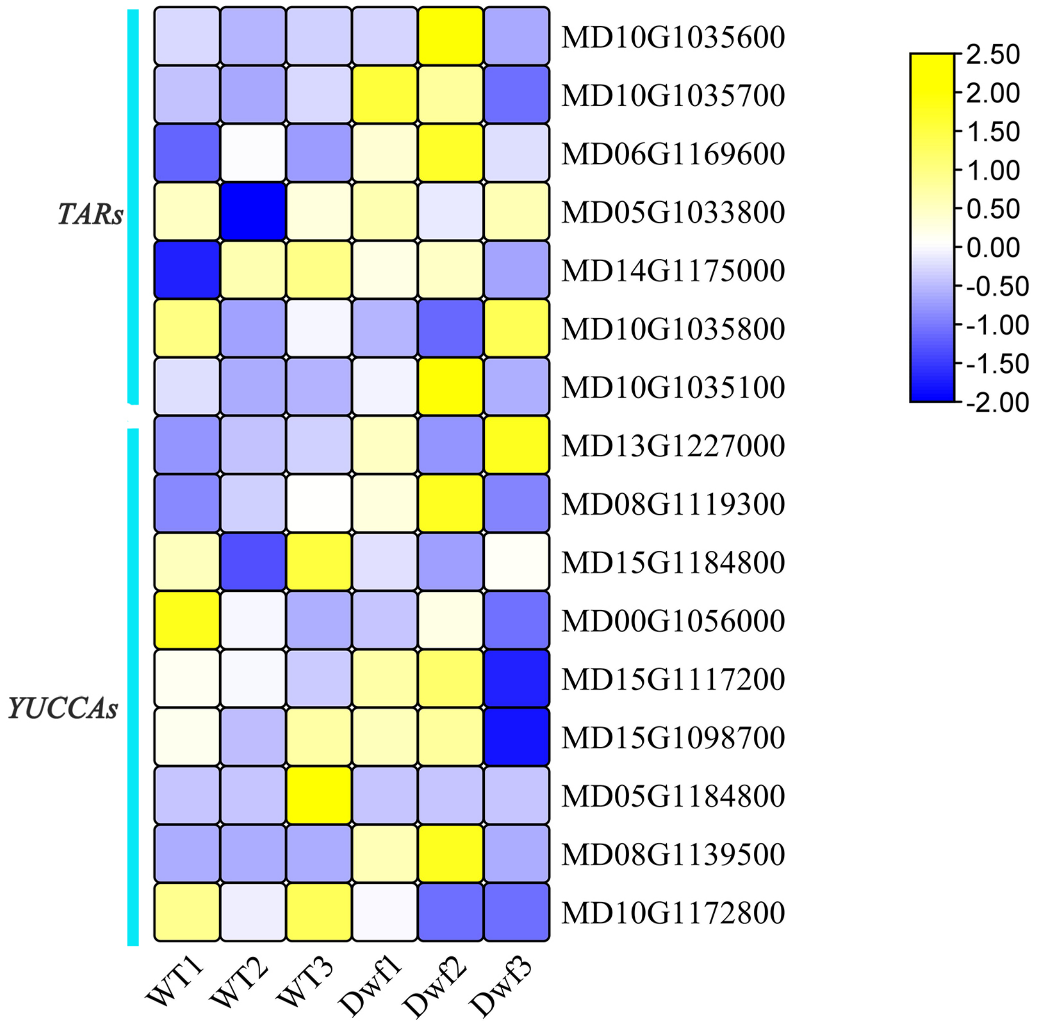

Figure S4. A heat map of seven TAR genes (tryptophan aminotransferase-related gene) and nine YUCCA genes (indole-3-pyruvate monooxygenase gene) in the WT and *Dwf* identified using RNA-seq.

Table S1. Primers used in this study

Purpose	Primer name	Sequence (5' to 3')
Primers for gene cloning	IAA19-CDS-F	ATGGCCAAAGAAGGTTTAGGG
	IAA19-CDS-R	TTATGGCTCCTCTTCCATTGTT
RNAi-IAA19	IAA19-forward-F	gagaacacgggggactctagaGCCAAAGAAGGTTTAGGGCTTG
	IAA19-forward-R	tggATTCTTCAACACCTCGCCGA
	Intron-F	gcgaggtgttgaagaatCCAATTGGTAAGGAAATAATTATTTTCT
	Intron-R	cggcgaggtgttgaagaatATCGATTTCGAACCCAGCTTC
	IAA19-anti-F	tATTCTTCAACACCTCGCCGA
	IAA19-anti-R	cgatcggggaaattcgagctcGCCAAAGAAGGTTTAGGGCTTG
Subcellular localization	IAA19GFP-F	agctcgggtacccggggatccATGGCCAAAGAAGGTTTAGGG
	IAA19GFP-R	ccttgctcaccatggtgtcgacTGGCTCCTCTTCCATTGTT
GUS activity assays	1391-proIAA19-F	tggctgcaggtcgacggatccGTTTAAGTTTTTTTGCTGTTAATTTAAA A
	1391-proIAA19-R	tcttagaattcccggggatccTTGGGTTTTGAGGGTTTTGAGA
Relative expression level	IAA19 -F	CAGATCTTGCTCTGGCGTTG
	IAA19 -R	TTGTGCTGCAGTCCAAATCC
	18S-F	TGACCGAATGAGCAAGGAAATTACT
	18S-R	TACTCAGCTTTGGCAATCCACATC
	SAUR-12-F	ATGCTCGAGCTTGGGAAAGA
	SAUR-12-R	CAAATTCCTCTTCGGCTTGG
	ARP1-F	AGAGTTGGGCTGTGGCTTGT
	ARP1-R	TTCCGCTCGTGCTAAAGACA
	LBD14-F	GCTCTCCGATTCCTTGCTGT
	LBD14-R	CCAACAAAGTAGGCTCCGGT
	SF21-1-F	TAATGACAGCAACCGATCCA
	SF21-1-R	CCCATGTTTGGCAGAGTTTT
	PAP17-F	CCCCACCAAACAAGATGGAT
	PAP17-R	GTCATGCTCGCTGGTGAGTC
	ERF1B-F	GGGCACCCAAGAAATCACAT
	ERF1B-R	GCCTAGCCACACCCTTATGC
	bHLH18-F	AAGGCCAGTCAGGGAACAAA
	bHLH18-R	TCACAAACACGACCGATTCC
	bHLH10-F	CCGAGGTTGATATCCGCATT
	bHLH10-R	ACTGCCGGTAGGTGGAACTG
	MYB4-F	GCAAGAGCTAGACCCCGTTG
	MYB4-R	CATCAGAATTCCCGGCTGAT
	TCP9-F	ATAGCCACAGGCACCATTCC
	TCP9-R	CGACGCAACTCCACTACGAC
	EBF1-F	GTGCCCGTTGGTATCTGACA
	EFB1-R	TCCCGATCACTGTGGTTGAC
	CYP90C1-F	GGCTACACCTCTCGACCTGTCAG
	CYP90C1-R	CCACCTTGTTCACCTCAGCATCC

SAUR-51b-F	AGCAGCAACACTACGACGAACAG
SAUR-51b-R	CGGGTCAGGAAGGAGATCGGTAC
SAUR-51a-F	CAGTTCCAATGCCTCCGCCAAG
SAUR-51a-R	GACTTCTTCGCAAGGGATGGTGAG
SAUR-14b-F	GTGAGAAGCAGCGGTTTGTTGTTC
SAUR-14b-R	CGCCCATTGGGTGATCATATCCG
SAUR-14a-F	AGAAGCAGCGGTTCGTTGTTCC
SAUR-14a-R	TTCCTCCTCAGCGTCACTCAGC
BKI1-F	CTCCGACGAATAGTGGGCATCTTG
BKI1-R	GGGCTTCTCTTCTGTGGCAATGG
